# SOD2 Mediates Curcumin-Induced Protection against Oxygen-Glucose Deprivation/Reoxygenation Injury in HT22 Cells

**DOI:** 10.1155/2019/2160642

**Published:** 2019-09-29

**Authors:** Yuqing Wang, Yuanyuan Zhang, Liang Yang, Jin Yuan, Ji Jia, Shuai Yang

**Affiliations:** ^1^Department of Rehabilitation, General Hospital of Southern Theatre Command of PLA, Guangzhou 510010, China; ^2^Department of 1st Geriatrics, General Hospital of Southern Theatre Command of PLA, Guangzhou 510010, China; ^3^Department of Cardiology, General Hospital of Southern Theatre Command of PLA, Guangzhou 510010, China; ^4^Department of Pharmacology, General Hospital of Southern Theatre Command of PLA, Guangzhou 510010, China; ^5^Department of Anesthesiology, General Hospital of Southern Theatre Command of PLA, Guangzhou 510010, China; ^6^Department of Neurosurgery, The First Affiliated Hospital of Sun Yat-sen University, Guangzhou 510080, China

## Abstract

Curcumin (Cur) induces neuroprotection against brain ischemic injury; however, the mechanism is still obscure. The aim of this study is to explore the potential neuroprotective mechanism of curcumin against oxygen-glucose deprivation/reoxygenation (OGD/R) injury in HT22 cells and investigate whether type-2 superoxide dismutase (SOD2) is involved in the curcumin-induced protection. In the present study, HT22 neuronal cells were treated with 3 h OGD plus 24 h reoxygenation to mimic ischemia/reperfusion injury. Compared with the normal cultured control group, OGD/R treatment reduced cell viability and SOD2 expression, decreased mitochondrial membrane potential (MMP) and mitochondrial complex I activity, damaged cell morphology, and increased lactic dehydrogenase (LDH) release, cell apoptosis, intracellular reactive oxygen species (ROS), and mitochondrial superoxide (*P* < 0.05). Meanwhile, coadministration of 100 ng/ml curcumin reduced the cell injury and apoptosis, inhibited intracellular ROS and mitochondrial superoxide accumulation, and ameliorated intracellular SOD2, cell morphology, MMP, and mitochondrial complex I activity. Downregulating the SOD2 expression by using siRNA, however, significantly reversed the curcumin-induced cytoprotection (*P* < 0.05). These findings indicated that curcumin induces protection against OGD/R injury in HT22 cells, and SOD2 protein may mediate the protection.

## 1. Introduction

Stroke is one of the leading causes of disability and death in China and worldwide [1]. In 2015, the number of new patients with stroke was more than 13 million, leading to a cost of 11.3 billion USD, which brought about great economic burden to the patients and the country [2]. However, at present, the effective neuroprotective drug against brain ischemic injury is very limited. Recombinant tissue plasminogen activator (rTPA) is the only neuroprotectant used in clinic; the limited therapeutic time window (within 4.5 h after the onset of stroke) reduces its utilization rate, leading to the result that only 3% to 8.5% of stroke patients can receive rTPA treatment [3, 4]. Therefore, exploring novel neuroprotective medicine against brain ischemic injury is very urgent and important.

Curcumin is derived from seasoning curry and herbal *Carcuma longa* Linn (turmeric), and some latest investigations showed that curcumin protects neuronal cells against brain ischemic injury both in vivo and in vitro [5, 6]. The curcumin-induced protection against ischemic injury, however, is still not clear. Type-2 superoxide dismutase (SOD2) is an antioxidative protein, which is expressed in mitochondria of cells, and the upregulation of SOD2 in cells induces neuroprotective effects [7, 8]. And some latest investigations indicated that neuronal oxidative injury and mitochondrial dysfunction are involved in the pathophysiological process of brain ischemic injury [9–11]. In addition, one of our studies showed that SOD2 protein mediates curcumin-induced protection against *β*-amyloid (A*β*) in neuronal cells [12].

Therefore, in the present study, we used oxygen-glucose deprivation plus reoxygenation (OGD/R) in HT22 neuronal cells to mimic neuronal ischemia/reperfusion (I/R) injury [13] and investigated whether SOD2 mediates curcumin-induced protection against OGD/R.

## 2. Materials and Methods

### 2.1. Materials

HT22 cells were obtained from the Xuzhou Medical University. Curcumin, Dulbecco's Modified Eagle Medium (DMEM), fetal bovine serum (FBS), dimethyl sulfoxide (DMSO), and methylthiazolyldiphenyl-tetrazolium bromide (MTT) were purchased from Sigma-Aldrich (St. Louis, MO, USA). Lactic dehydrogenase (LDH) reagent kit was obtained from Nanjing Jiancheng Bioengineering Institute (Nanjing, China). SOD2 activity assay kit was obtained from Trevigen (Gaithersburg, USA). The DAPI staining solution and ROS reagent kit were purchased from Beyotime Technology (Nantong, China). MitoSOX staining kits were purchased from Invitrogen Molecular Probes (San Diego, CA). The primary antibodies of anti-SOD2, anticleaved caspase-3, and anti-*β*-actin were purchased from Abcam (Cambridge, UK).

### 2.2. Cell Culture and OGD Treatment

The HT22 cells were cultured in the medium, containing 90% DMEM medium, 10% FBS, 100 U/ml penicillin, and 100 *μ*g/ml streptomycin. The air of the incubator contained 95% O_2_ and 5% CO_2_, and the temperature of the incubator was 37°C. The medium was changed every 2 to 3 days, and the cells were passaged 3 times/week with a 1:4 split ratio. We followed the methods of Du et al. [12].

For the OGD treatment, the medium of the cells was changed with DMEM medium without glucose and FBS, and then the cells were cultured in a sealed container; the air of the container contained 95% N_2_ and 5% CO_2_, and the temperature of the container was 37°C. After 3 h OGD treatment, the medium of the cells was changed with normal medium, and the cells were returned to the normal incubator to mimic reperfusion.

### 2.3. Experimental Protocols

**Figure 1:**
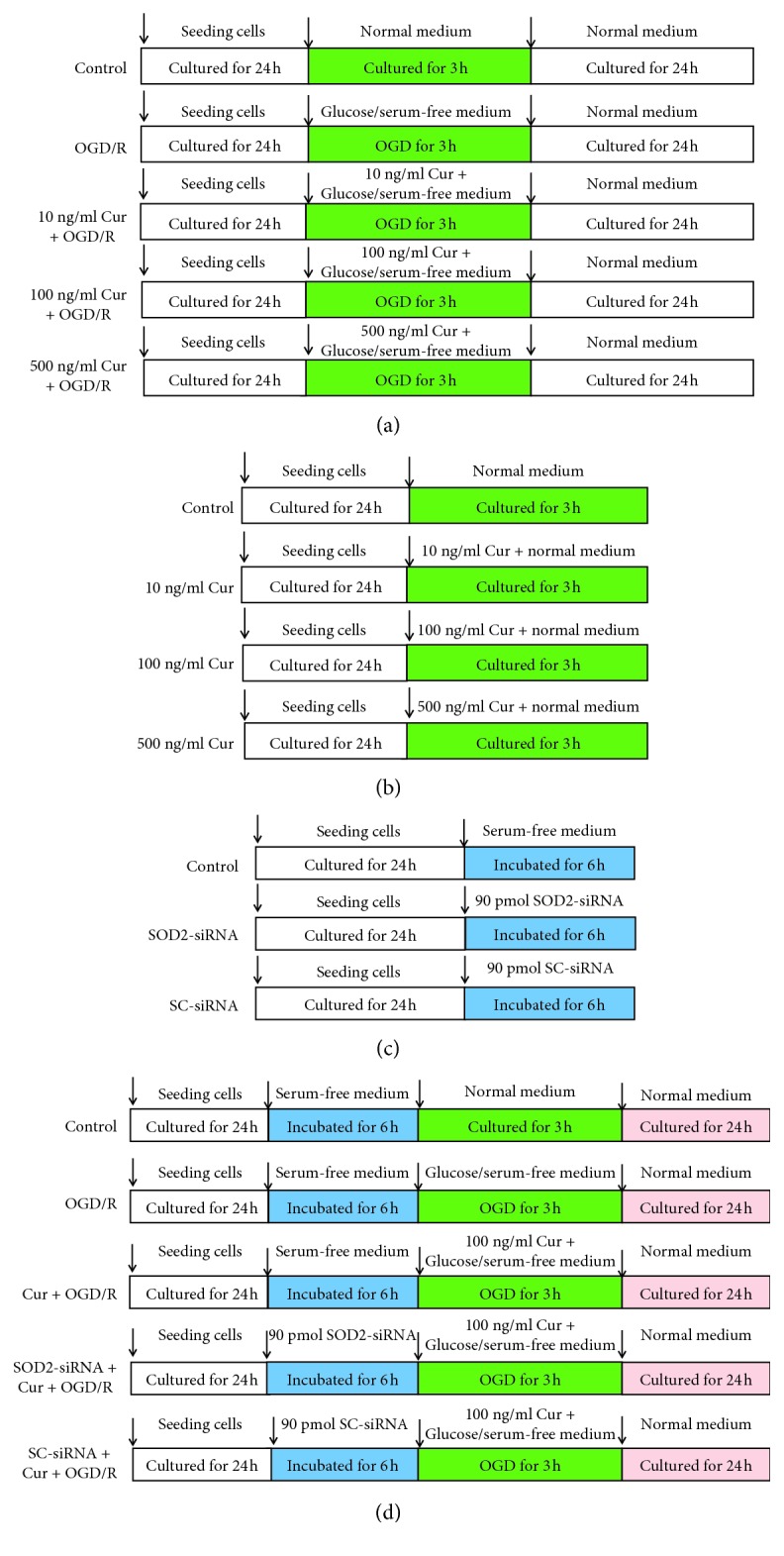
Experimental protocol diagram. (a) Searching a suitable curcumin concentration. The cells were divided into 5 groups, including the normal cultured control group, oxygen glucose 3 h OGD plus 24 reoxygenation treatment group (OGD/R), and 3 concentrations of curcumin treatment groups. After the treatments, cell viability and LDH release were assessed. (b) Observing curcumin-induced effect on SOD2 protein expression. The cells were divided into 4 groups, including normal cultured control group and 3 concentrations of curcumin treatment groups; after 3 h treatment, SOD2 expression was assessed by using western blot analysis. (c) Evaluating interfering effect of SOD2-siRNA. The cells were divided into 3 groups, including control group, SOD2-siRNA treatment group, and scrambled (SC)-siRNA treatment group. After the treatments, SOD2 protein expression was evaluated by using western blot analysis. (d) Exploring the role of SOD2 in curcumin-induced protection in HT22 cells. The cells were divided into 5 groups, including control, OGD/R treatment group, Cur + OGD/R group, SOD2-siRNA + Cur + OGD/R group, and SC-siRNA + Cur + OGD/R group; after the treatments, cell injury, apoptosis, SOD2 expression, cell morphology, intracellular ROS, mitochondrial functions, and superoxide were assessed.

To explore a suitable curcumin concentration, the cells were divided into 5 groups, including the normal cultured control group, 3 h OGD plus 24 h reoxygenation (OGD/R) treatment group, 10 ng/ml curcumin treatment group (10 ng/ml Cur + OGD/R), 100 ng/ml curcumin treatment group (100 ng/ml Cur + OGD/R), and 500 ng/ml curcumin treatment group (500 ng/ml Cur + OGD/R). After treatments (Figure 1(a)), cell viability and LDH release were assessed. Then, to observe curcumin-induced effect on SOD2 expression in normal cultured cells (Figure 1(b)), the cells were divided into 4 groups, including control and 3 concentrations of curcumin treatment groups (10, 100, and 500 ng/ml curcumin); after 3 h treatment, SOD2 expression was assessed. Next, to evaluate the SOD2-siRNA-induced effects on SOD2 expression and cytotoxicity, the cells were divided into 3 groups, including the normal cultured control group, SOD2-siRNA treatment group, and scrambled-siRNA (SC-siRNA) treatment group; after 6 h incubation, western blot and MTT assay were taken to evaluate SOD2 expression and cell injury level (Figure 1(c)).

To determine whether SOD2 mediates curcumin-induced protection against OGD/R in HT22 cells, the cells were divided into 5 groups, including control group, OGD/R treatment group, 100 ng/ml curcumin treatment group (Cur + OGD/R), SOD2-siRNA treatment group (SOD2-siRNA + Cur + OGD/R), and SC-siRNA treatment group (SC-siRNA + Cur + OGD/R). After the treatments (Figure 1(d)), cell injury, apoptosis, SOD2 expression, cell morphology, mitochondrial functions, intracellular ROS, and mitochondrial superoxide were assessed.

### 2.4. Cell Viability

The HT22 cells were seeded into a 96-well cell culture plate at a density of 1 × 10^5^ cells per well. After the treatments, 20 *μ*l MTT solution (5 *μ*g/ml) was added into each well; after 4 h incubation at 37°C, the medium of the cell culture plate was removed. Then, 150 *μ*l DMSO was added into each well. After 15 min shaking, as the formazan was dissolved completely, the absorbance of each well was measured by using a spectrophotometer (TECAN, CH).

### 2.5. LDH Release

The cells were seeded into a 24-well cell culture plate at a density of 5 × 10^5^ cells/well. After the treatments, the supernatants of the plate were collected to measure the LDH activity of each well, as previously described [14].

### 2.6. Western Blot Analysis

The cells were seeded into a 6-well cell culture plate at a density of 1 × 10^6^ cells/well. After the treatments, the medium was removed, and the cells were collected. Then, the total protein of the treated cells was evaluated by using the Bradford method as described previously [14]. The primary anti-SOD2 (1 : 1000 in dilution), anticleaved caspase-3 (1 : 50 in dilution), and anti-*β*-actin (1 : 1000 in dilution) antibodies were used. The antigens were assessed by using the chemiluminescence technique (Amersham Pharmacia Biotech Piscataway, USA). Image analysis was evaluated with the computerized analysis software (Bio-Rad, Hercules, CH). We followed the methods of Du et al. [12].

### 2.7. siRNA Interfering

SOD2-siRNA and SC-siRNA were obtained from Qiangen (Germany). The siRNA oligomers, including SOD2-siRNA and SC-siRNA, were diluted in serum-free DMEM medium, and then the medium was incubated in room temperature for 5 min. The incubated oligomers were combined with diluted Lipofectamine 2000 and incubated for another 20 min. The cell culture medium was then removed from the plate, and the cells were washed twice with phosphate-buffered saline (PBS) at 37°C. Then, the complexes of 90 pmol siRNA and Lipofectamine were added into the cell culture plate, and the cells were incubated for 6 h in the incubator at 37°C. Then, the cells were washed with PBS at 37°C. The SOD2 expression was measured using western blot analysis.

### 2.8. SOD2 Activity Evaluation

HT22 cells were seeded into a 6-well cell culture plate at a density of 1 × 10^6^ cells/well. After the treatments, as previously described in detail [15], SOD2 activities in U/mg protein were calculated.

### 2.9. Cell Apoptosis Evaluation

The cell apoptosis level was measured by using a flow cytometry (BD, USA). The cells were planted into a 6-well cell culture plate at a density of 5 × 10^5^ cells/well. After the treatments, the cells were harvested by centrifugation at 1000 rpm for 10 min. Then, the supernatants of the cells were removed, and the cells were washed twice by ice-cold PBS. After the washing, the apoptotic rates of the cells were evaluated as previously described [12, 14].

### 2.10. Mitochondrial Function Evaluation

The mitochondria of the treated cells were isolated by using a mitochondrial isolation kit according to the manufacturer's instructions (Qiagen, Hilden, Germany). And the mitochondrial complex I activity was measured at 30°C as previously described by Han et al. [16]. In brief, the mitochondrial complex I activity of HT22 cells was measured by following rotenone-sensitive oxidation of NADH initiated by ubiquinone-1 (Q1). An appropriate amount of cell lysate was added into 0.5 ml assay mixture, containing 20 mM potassium phosphate buffer, 2 mM NaN_3_, 0.15 mg/ml phospholipid, 0.1 mM Q1, and 0.15 mM NADH, and the pH value was 8.0. Mitochondrial complex I activity was evaluated by assessing the decrease in absorbance (340 nm) and confirmed by inhibition with 40 *μ*M rotenone. And the activity level (nmol NADH oxidized·min^−1^ mg·protein^−1^) was calculated with a molar extinction coefficient of 6.22 mM^−1^ cm^−1^.

Mitochondrial membrane potential (MMP) of the treated HT22 cells was assessed by using the JC-1 (Sigma-Aldrich, St. Louis, MO, USA). According to the manufacturer's instruction, mitochondrial samples were exposed to JC-1 staining buffer. At the end of the experiments, valinomycin was used as the negative control. And the fluorescence intensity of the cells was measured by using a fluorescence spectrophotometer (TECAN, CH), and the measurement temperature was 37°C. The ratio of aggregates to monomer was calculated as the MMP indicator, and the wavelengths testing the aggregates and monomer were 590 nm (red) and 525 nm (green), respectively. We followed the methods of Du et al. [12].

### 2.11. Cell Morphology Observation

The cells were planted into a 6-well cell culture plate at a density of 5 × 10^5^ cells/well. After the treatments, the cells were observed by using a phase-contrast microscope, and the photos of the cells were taken randomly.

### 2.12. Intracellular Reactive Oxygen Species

HT22 cells were seeded into a confocal microscopy special dish at a density of 2 × 10^5^ cells/well. After the treatments, a reactive oxygen species (ROS) assay kit (Beyotime Technology, Nantong, China) was taken to evaluate the intracellular ROS level. In brief, the DMEM medium without FBS was added into each well, containing 100 *μ*M DCF-DA (nonfluorescent and colorless). After 20 min incubation at 37°C, the DCF-DA was oxidized into the fluorescent 2′,7′-dichlorofluorescein (DCF) by intracellular ROS. The dish was washed three times with PBS, and then the photos were taken by using a confocal microscope (excitation = 480 nm; emission = 535 nm). Finally, the fluorescence intensities of the photos were evaluated by using Image-Pro Plus software.

### 2.13. Mitochondrial Superoxide Assay

MitoSOX reagent was used to measure mitochondrial superoxide level. In brief, the cells were seeded into a confocal microscopy special dish at a density of 1 × 10^5^ cells/well. After the treatments, the HT22 cells were treated with 5 *μ*M MitoSOX reagent for 20 min at 37°C; at the end of the treatments, 100 *μ*l DAPI staining solution was added into the dish to mark the cell nuclei. After being washed three times with PBS, a confocal microscope was used to observe and take fluorescence photos of the cells, including mitochondrial superoxide (red, excitation = 510 nm; emission = 580 nm) and nuclei (blue, excitation = 340 nm; emission = 488 nm). Then, the fluorescence intensity of the mitochondrial superoxide was calculated by using Pro-plus software (IPP 6.0, Media Cybernetics, Silver Spring, MD, USA).

### 2.14. Statistical Analysis

The data of this study were analyzed by using SPSS 13.0 software (SPSS Inc., Chicago, USA). The values of all the experiments were expressed as means ± standard deviation (SD), and one-way ANOVA was used to assess the data. Tukey's multiple comparison was taken to compare the differences between the groups. *P* < 0.05 indicated statistical significance.

## 3. Results

### 3.1. Curcumin Reduced Cell Injury in OGD/R-Treated HT22 Cells and Upregulated SOD2 Expression

**Figure 2:**
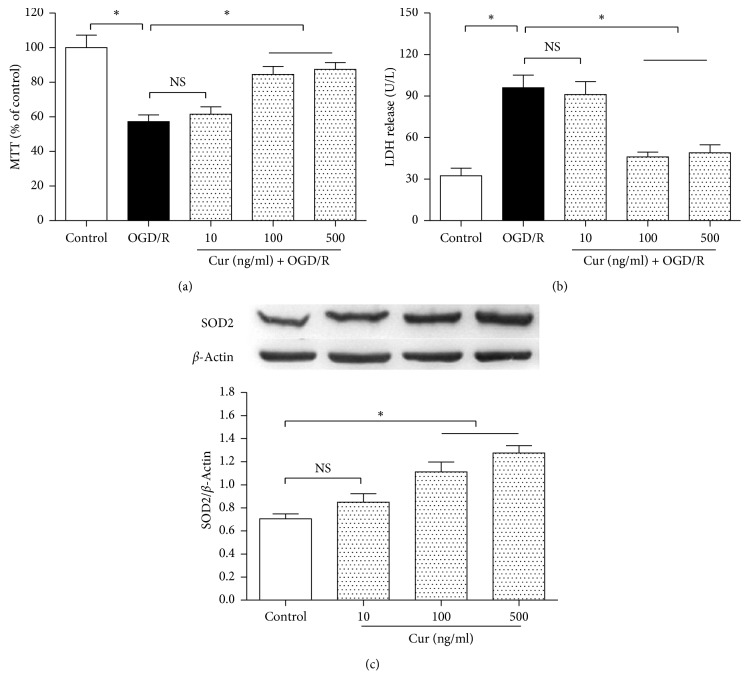
Curcumin decreased cell injury in HT22 cells exposed to OGD/R and upregulated SOD2 expression in normal condition. The HT22 cells were divided into 5 groups, including control, OGD/R, and 3 concentrations (10 ng/ml, 100 ng/ml, and 500 ng/ml) of curcumin plus OGD/R groups. After the treatments, cell viability and LDH release were measured by using the MTT method and reagent kit, respectively. Then, the cells were divided into 4 groups, including control and 3 concentrations (10 ng/ml, 100 ng/ml, and 500 ng/ml) of curcumin treatment groups; after 3 h exposure, western blot was performed to assess SOD2 expression. (a) Curcumin restored cell viability (*n* = 8). (b) Curcumin reduced LDH release (*n* = 8). (c) Curcumin increased SOD2 expression (*n* = 4). Results are expressed as means ± SD. ^*∗*^*P* < 0.05; NS: no significance.

To find a suitable curcumin (Cur) treatment concentration, the HT22 cells were divided into 5 groups, including control, OGD/R, and 3 concentrations of curcumin treatment groups (10, 100, and 500 ng/ml curcumin plus OGD/R respectively). After 3 h OGD and 24 h reoxygenation treatment, compared with the control, OGD/R treatment reduced cell viability (Figure 2(a)) and increased LDH activity (Figure 2(b)) in the medium significantly (*P* < 0.05), and 100 and 500 ng/ml curcumin treatment restored cell viability and decreased LDH activity obviously (*P* < 0.05). Then, the cells were divided into 4 groups (Figure 2(c)), including control and 3 doses of curcumin treatment groups (10, 100 and 500 ng/ml curcumin). After 3 h treatment, compared with the control group, 100 and 500 ng/ml curcumin groups showed significantly increased SOD2 expression (*P* < 0.05). The curcumin concentration of 100 ng/ml was used in the subsequent experiments.

### 3.2. Downregulation of SOD2 Expression Reversed Curcumin-Induced Effects on Cell Injury, SOD2 Expression, and Activity

**Figure 3:**
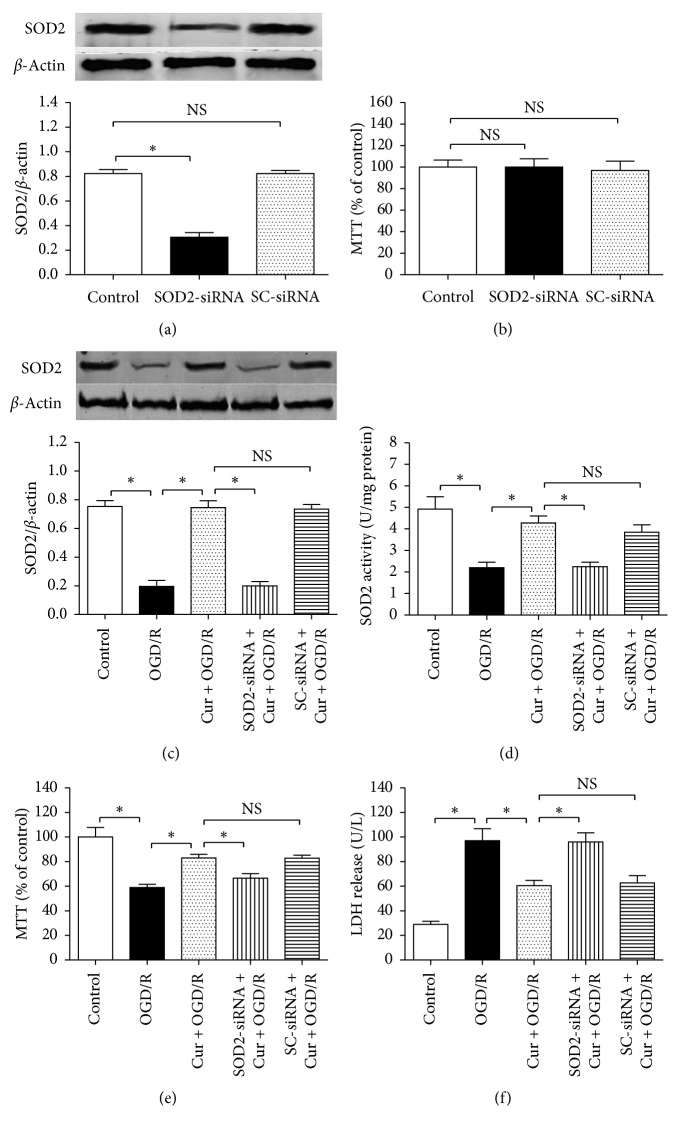
SOD2-siRNA reversed curcumin-induced cytoprotection and SOD2 upregulation in HT22 cells exposed to OGD/R. The cells were divided into 3 groups, including control, SOD2-siRNA, and scrambled (SC)-siRNA; after 6 h incubation, western blot and MTT assay were taken to assess SOD2 expression and cell viability, respectively. Then, the cells were divided into 5 groups, including control, OGD/R, Cur + OGD/R, SOD2-siRNA + Cur + OGD/R, and SC-siRNA + Cur + OGD/R; after 3 h OGD plus 24 h reoxygenation, SOD2 expression and activity, cell viability, and lactic dehydrogenase (LDH) were assessed. (a) SOD2-siRNA inhibited SOD2 protein expression (*n* = 4). (b) Either SOD2-siRNA or SC-siRNA induced no obvious cytotoxicity (*n* = 8). (c, d) SOD2-siRNA reversed curcumin-induced effects on SOD2 expression (*n* = 4) and activity (*n* = 8). (e) SOD2-siRNA reversed curcumin-induced cell viability restoration (*n* = 8). (f) SOD2-siRNA reversed curcumin-induced LDH release decrease (*n* = 8). Results are expressed as means ± SD. ^*∗*^*P* < 0.05; NS: no significance.

To explore the role of SOD2 in curcumin-induced protection against OGD/R in HT22 cells, SOD2-siRNA was taken to downregulate SOD2 protein expression (Figure 3(a)). The SOD2-siRNA used in this study reduced SOD2 expression significantly (0.31 ± 0.04 vs. 0.82 ± 0.03; *P* < 0.05), but the scrambled siRNA (SC-siRNA) did not reduce SOD2 expression (0.81 ± 0.03 vs. 0.82 ± 0.03; *P* > 0.05). Meanwhile, compared with the normal cultured control (Figure 3(b)), either the SOD2-siRNA or the SC-siRNA induced no obvious cytotoxicity (*P* > 0.05).

Then, the cells were divided into 5 groups, including control, OGD/R, Cur + OGD/R, SOD2-siRNA + Cur + OGD/R, and SC-siRNA + Cur + OGD/R. Compared with the control, 3 h OGD plus 24 h reoxygenation treatment (OGD/R) decreased SOD2 protein expression, SOD2 activity and cell viability, and increased LDH activity in the medium; concurrently, coadministration with 100 ng/ml curcumin restored SOD2 expression, SOD2 activity, and cell viability and reduced LDH release (Figures 3(c)–3(e)). However, the SOD2-siRNA, but not the SC-siRNA (*P* > 0.05), abolished the curcumin-induced cytoprotective effects against OGD/R injury significantly (*P* < 0.05). These findings showed that SOD2 mediates curcumin-induced protective effects against OGD/R in HT22 cells.

### 3.3. Downregulation of SOD2 Expression Reversed Curcumin-Induced Inhibitions of Cell Apoptosis and Cleaved Caspase-3 Expression

**Figure 4:**
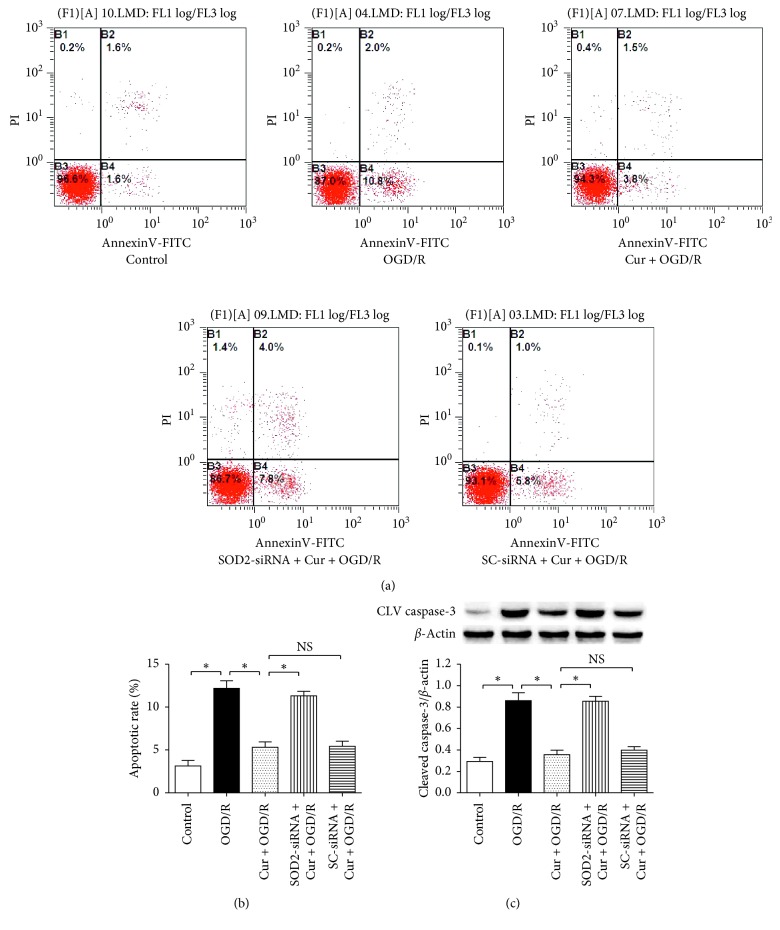
SOD2-siRNA reversed curcumin-induced antiapoptotic effects in HT22 cells exposed to OGD/R. The cells were divided into 5 groups, including control, OGD/R, Cur + OGD/R, SOD2-siRNA + Cur + OGD/R, and SC-siRNA + Cur + OGD/R; after 3 h OGD and 24 h reoxygenation, cell apoptotic rate and cleaved caspase-3 expression were evaluated by using flow cytometry and western blot, respectively. (a) Flow cytometry results of cells. (b) SOD2-siRNA reversed curcumin-induced antiapoptotic effect (*n* = 6). (c) SOD2-siRNA reversed curcumin-induced downregulation of cleaved caspase-3 expression (*n* = 4). Results are expressed as means ± SD. ^*∗*^*P* < 0.05; NS: no significance.

To investigate the curcumin-induced antiapoptosis in HT22 cells exposed to OGD/R, flow cytometry and western blot were used to evaluate cell apoptotic rate and apoptosis-associated protein cleaved caspase-3 expression (Figures 4(a)–4(c)). Compared with the control, the OGD/R treatment increased cell apoptosis and cleaved caspase-3 expression (*P* < 0.05), and 100 ng/ml curcumin reduced cell apoptosis and cleaved caspase-3 expression level (*P* < 0.05); similarly, SOD2-siRNA, but not the SC-siRNA (*P* > 0.05), markedly abolished the curcumin-induced antiapoptotic effects above (*P* < 0.05). These results indicated that SOD2 protein mediates curcumin-induced antiapoptosis in HT22 cells treated with OGD/R.

### 3.4. Downregulation of SOD2 Expression Abolished Curcumin-Induced Ameliorations of Cell Morphology and Mitochondrial Functions

**Figure 5:**
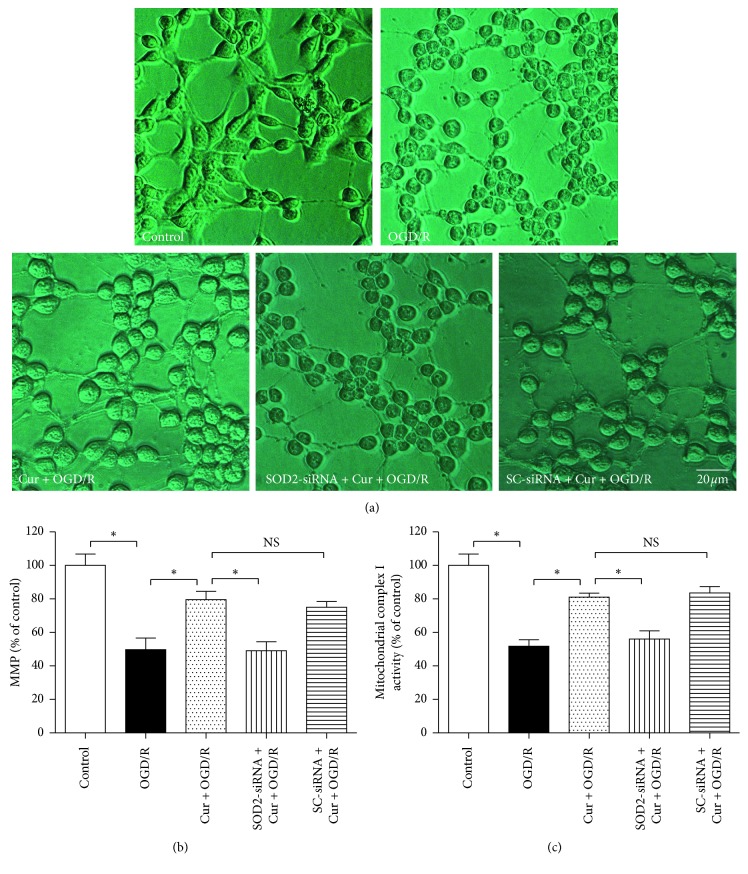
SOD2-siRNA reversed curcumin-induced ameliorations of cell morphology and mitochondrial functions in HT22 cells exposed to OGD/R. The cells were divided into 5 groups, including control, OGD/R, Cur + OGD/R, SOD2-siRNA + Cur + OGD/R, and SC-siRNA + Cur + OGD/R; after 3 h OGD and 24 h reoxygenation, cell morphology and mitochondrial functions were evaluated. (a) SOD2-siRNA reversed curcumin-induced cell morphology amelioration. (b) SOD2-siRNA reversed curcumin-induced amelioration of mitochondrial membrane potential (MMP) (*n* = 8). (c) SOD2-siRNA reversed curcumin-induced amelioration of mitochondrial complex I activity (*n* = 8). Results are expressed as means ± SD. ^*∗*^*P* < 0.05; NS: no significance; Bar = 20 *μ*m.

To further observe the curcumin-induced effects on cell morphology and mitochondrial functions in HT22 cells exposed to OGD/R, phase-contrast microscope and reagent kits were taken to assess cell morphology and cellular mitochondrial functions. The cells were divided into 5 groups as shown in the Figure 5. Compared with the control, 3 h OGD plus 24 h reoxygenation (OGD/R) treatment damaged the cellular morphology (Figure 5(a)) and reduced mitochondrial membrane potential (MMP) and mitochondrial complex I activity (*P* < 0.05), which are associated with the mitochondrial functions (Figures 5(b) and 5(c)); 100 ng/ml curcumin maintained cell integrity and restored MMP and mitochondrial complex I activity (*P* < 0.05). However, the SOD2-siRNA, but not the SC-siRNA (*P* > 0.05), reversed the curcumin-induced protection in cell morphology and mitochondrial functions (*P* < 0.05). These findings showed that SOD2 mediates curcumin-induced protection in cell morphology and mitochondrial functions against OGD/R in HT22 cells.

### 3.5. Downregulation of SOD2 Expression Inhibited Curcumin-Induced Ameliorations on Intracellular ROS and Mitochondrial Superoxide

**Figure 6:**
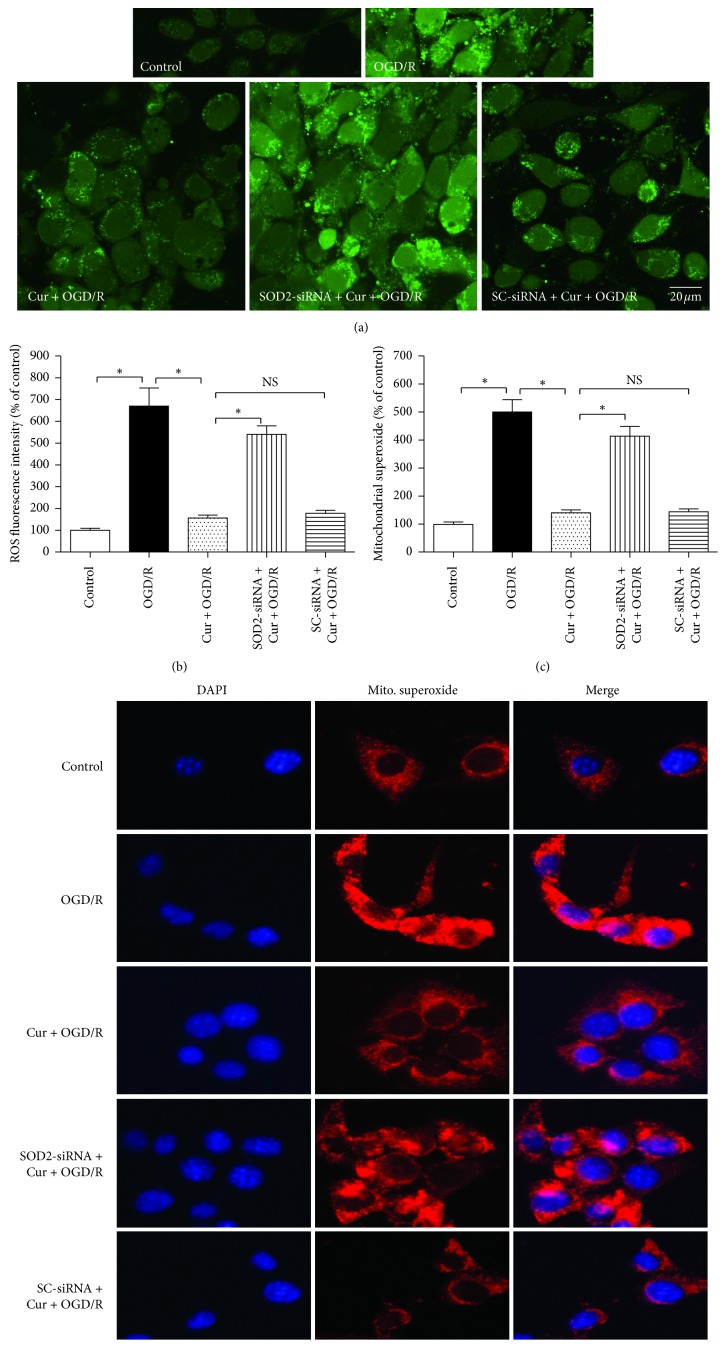
SOD2-siRNA reversed curcumin-induced reduction of intracellular ROS and mitochondrial superoxide in HT22 cells exposed to OGD/R. The cells were divided into 5 groups, including control, OGD/R, Cur + OGD/R, SOD2-siRNA + Cur + OGD/R, and SC-siRNA + Cur + OGD/R; after 3 h OGD and 24 h reoxygenation, intracellular ROS and mitochondrial superoxide were evaluated. (a) Intracellular ROS fluorescence staining results. (b) SOD2-siRNA reversed curcumin-induced intracellular ROS reduction (*n* = 8). (c) SOD2-siRNA reversed curcumin-induced mitochondrial superoxide reduction (*n* = 8). (d) Mitochondrial superoxide fluorescence staining results. Results are expressed as means ± SD. ^*∗*^*P* < 0.05; NS: no significance; Bar = 20 *μ*m.

High level of intracellular ROS and mitochondrial superoxide can damage cell and mitochondria. Compared with the control group, 3 h OGD plus 24 reoxygenation (OGD/R) treatment increased ROS (Figures 6(a) and 6(b)) and mitochondrial superoxide levels (Figures 6(c) and 6(d)), and coadministration with 100 ng/ml curcumin reduced the ROS and mitochondrial superoxide levels obviously (*P* < 0.05); similarly, SOD2-siRNA, but not the SC-siRNA (*P* > 0.05), reversed the curcumin-induced downregulation on intracellular ROS and mitochondrial superoxide (*P* < 0.05). These observations showed that SOD2 mediates curcumin-induced antioxidation effects against OGD/R in HT22 cells.

## 4. Discussion

**Figure 7:**
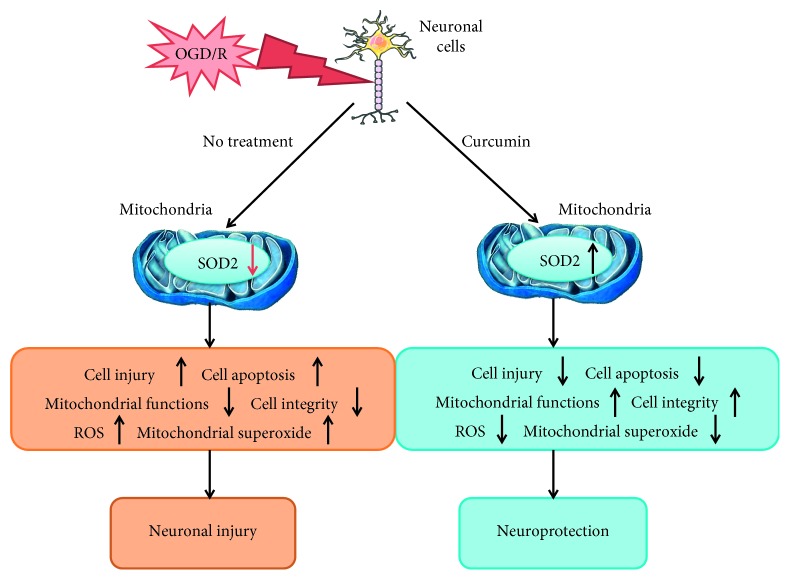
Curcumin induces neuroprotection against OGD/R in neuronal cells via upregulating SOD2 protein. Oxygen-glucose deprivation and reoxygenation (OGD/R) injury can downregulate SOD2 expression, increase intracellular ROS and mitochondrial superoxide accumulations, then damage neuronal cells, increase cell apoptosis, cause mitochondrial dysfunctions, and undermine cell integrity, leading to neuronal injury ultimately. Coadministration of curcumin, however, could upregulate SOD2 expression, reduce intracellular ROS and mitochondrial superoxide accumulations, and ameliorate mitochondrial functions and cell integrity, causing neuroprotection.

In the present study, the HT22 neuronal cells were exposed to OGD for 3 h and then cultured in normal medium for 24 h to imitate the neuronal I/R injury, and 100 ng/ml curcumin was coadministered to reduce the OGD/R-induced cell injury. Compared with the control, OGD/R increased the cell injury, apoptosis, intracellular ROS, and mitochondrial superoxide, reduced mitochondrial functions and intracellular SOD2, and damaged cell morphology; meanwhile, the presence of curcumin reduced cell injury, apoptosis, intracellular ROS, and mitochondrial superoxide, restored mitochondrial functions and SOD2, and maintained cell integrity. However, SOD2-siRNA, but not the SC-siRNA, significantly reversed the curcumin-induced protections above. These findings indicated that curcumin alleviates OGD/R-induced injury in HT22 cells, and SOD2 may mediate the curcumin-induced protection (Figure 7).

Stroke is one of the leading causes of disability and death in the worldwide [17]. Unfortunately, however, effective medicine or therapy in treating the disease is extremely limited. The limited therapeutic time window of rTPA decreases its use greatly. For this reason, exploring medicine or therapy for stroke is of great importance. According to the findings of some latest investigations, oxidative injury and mitochondrial dysfunction participate in the pathophysiological process of stroke [9–11]. Oxidative injury can consume intracellular antioxidants, including glutathione (GSH), catalase (CAT), and SOD, and also damage the neuronal membrane [18, 19]. Meanwhile, especially after reperfusion of stroke, too much ROS could be generated [20], which may injure mitochondria and consume SOD2 [21]. Therefore, ameliorating mitochondrial functions and increasing SOD2 level are regarded to be effective in treating stroke. As stroke causes ischemic injury and reperfusion injury to brain tissue, and reperfusion injury may be more serious than ischemic injury [22]. In this study, the HT22 cells were treated with 3 h OGD injury and then cultured in normal medium for 24 h to mimic the I/R injury of stroke. Apoptosis is an important cell death pattern after I/R injury, and to assess the cell apoptosis degree, we took flow cytometry and western blot to measure cell apoptosis rate and cleaved caspase-3 expression, which is an apoptosis-associated protein, and its expression level is closely related to the apoptosis of cells [23]. In this study, curcumin reduced cleaved caspase-3 expression in the OGD/R-treated HT22 cells, and SOD2-siRNA reversed the antiapoptosis effects of curcumin, indicating that the curcumin-induced antiapoptosis in neuronal cells exposed to OGD might be via SOD2 protein.

Curcumin is an extract from seasoning curry and herbal *Carcuma longa* Linn. Some recent investigations reported that curcumin can induce antioxidation, anticerebral infarction, anti-inflammation, and neuroprotection [24–26]. In addition, some other studies showed that curcumin can ameliorate mitochondrial functions in neuronal cells [27]. Xie et al. [28] reported that curcumin can reduce neuronal injury against I/R and OGD/R in vivo and in vitro, and restricting Bax activation may be the key neuroprotective mechanism of curcumin. In another two investigations, nuclear factor erythroid-2-related factor 2 (Nrf2), a nuclear transcription factor with neuroprotective effects against central nervous system disease, was reported to be involved in curcumin-induced neuroprotection against hypoxic-ischemic brain injury in neonatal rats and against OGD/R injury in primary cultured cortical neurons [29, 30]. In the present study, we explored the role of curcumin in OGD/R-induced neuronal injury and determined whether SOD2 protein mediates curcumin-induced potential protection against OGD/R. We found that curcumin alleviated the OGD/R-induced cell injury and apoptosis, maintained cell morphology and mitochondrial functions, and increased SOD2 expression, ROS, and mitochondrial superoxide in the cells. Downregulating SOD2 expression, however, obviously abolished the curcumin-induced cytoprotection and mitochondrial function improvement and also reversed the curcumin-induced reductions on intracellular ROS and mitochondrial superoxide. High-level ROS and mitochondrial superoxide accumulation can oxidize and damage cell membrane and mitochondria. SOD2 is a protein expressed in mitochondria. Too much consumption of SOD2 can damage mitochondria and inhibit mitochondrial functions, leading to a reduction in mitochondrial energy generation [31]. Then, without enough energy supply, general functions of neuronal cells, including proliferation, action potential, excitability, and activation, could be abnormal. For the neuronal cells, energy supply deficiency can increase glutamate (an excitatory neurotransmitter) release, and the neuronal excitability may be upregulated, and long-term exposure to glutamate can activate the N-methyl-D-aspartic acid (NMDA) receptors expressed in the neuronal membrane, causing neural death and dysfunction ultimately [32]. In this study, we measured the MMP and mitochondrial complex I levels in the cells. The activation of NMDA receptors can induce overload of intracellular calcium [32]. Then, high concentration of intracellular calcium may inhibit oxidative phosphorylation and MMP, and the ability of mitochondria to produce energy (ATP) will decrease, leading to chloride ion infusion and cell death ultimately [33]. Mitochondrial respiratory chain consists of five complexes, including mitochondrial complex I-V, which are also called mitochondrial complex enzymes, and complex I is the most sensitive respiratory enzyme to ischemia among the five. Mitochondrial complex I can oxidize tricarboxylic acid cycle-produced NADH in the inner mitochondrial membrane, and it participates in producing ATP with ATPase. And brain ischemia results in greater complex I injury after brain oxygen deprivation [34]. Therefore, we evaluated MMP and mitochondrial complex I levels in the OGD/R-treated cells to determine the protective effects of curcumin in mitochondrial functions. Except for SOD2, SOD1 and SOD3 also induce protection against oxidative injury, and SOD1 is expressed in intercellular space, while SOD3 is in the cytoplasm [35]. In addition, evaluating the change of mitochondrial function is another aim of this study. Therefore, we explored SOD2, but not SOD1 or SOD3. In one study about osteoblasts in mice, SOD2 protected mitochondria against oxidative injury and increased osteoblast differentiation [36]. Another study reported that curcumin increased intracellular SOD2 level in human hepatoma cells [37]. In one of our studies, we found that curcumin can protect HT22 cells against A*β*-induced injury, and SOD2 protein mediates the curcumin-induced protection [12]. The findings of the present study explained, to some extent, the neuroprotective mechanism of curcumin against brain I/R. However, there are several limitations in our investigation. In the first place, the findings of this study were from in vitro and neuronal cell line, and whether similar results can be observed in vivo or in primary cultured neurons is unknown. In addition, except for SOD2, whether SOD1 or SOD3 mediates curcumin-induced neuroprotection is also not clear.

Taken together, in the present study, we found that curcumin can reduce OGD/R-induced cell injury in HT22 cells, and SOD2 protein mediates the curcumin-induced neuroprotection.

## Data Availability

All datasets analyzed during the current study are available from the corresponding author upon reasonable request.
